# The Impact of Chronic Obstructive Pulmonary Disease Severity on Psychological and Functional Outcomes: A Cross-Sectional Analysis

**DOI:** 10.3390/jcm14061865

**Published:** 2025-03-10

**Authors:** Alexandru Florian Crisan, Camelia Corina Pescaru, Adelina Maritescu, Emil Robert Stoicescu, Vlad Carunta, Cristian Oancea

**Affiliations:** 1 Research Center for Assessment of Human Motion, Functionality, and Disability, “Victor Babes” University of Medicine and Pharmacy Timisoara, Eftimie Murgu Square 2, 300041 Timisoara, Romania; crisan@umft.ro; 2 Pulmonary Rehabilitation Center, Clinical Hospital of Infectious Diseases and Pulmonology, “Victor Babes”, Gheorghe Adam Street 13, 300310 Timisoara, Romania; adelina.maritescu@umft.ro (A.M.); carunta.vlad@gmail.com (V.C.); 3 Center for Research and Innovation in Personalized Medicine of Respiratory Diseases (CRIPMRD), “Victor Babes” University of Medicine and Pharmacy Timisoara, Eftimie Murgu Square 2, 300041 Timisoara, Romania; oancea@umft.ro; 4 Doctoral School, “Victor Babes” University of Medicine and Pharmacy Timisoara, Eftimie Murgu Square 2, 300041 Timisoara, Romania; stoicescu.emil@umft.ro; 5 Faculty of Mechanics, Field of Applied Engineering Sciences, Specialization Statistical Methods and Techniques in Health and Clinical Research, ‘Politehnica’ University Timisoara, Mihai Viteazul Boulevard No. 1, 300222 Timisoara, Romania; 6 Research Center for Pharmaco-Toxicological Evaluations, “Victor Babes” University of Medicine and Pharmacy Timisoara, Eftimie Murgu Square No. 2, 300041 Timisoara, Romania; 7 Pulmonology Clinic, Clinical Hospital of Infectious Diseases and Pulmonology, “Victor Babes”, Gheorghe Adam Street 13, 300310 Timisoara, Romania

**Keywords:** COPD, psychological factors, functional outcomes, disease severity, self-compassion

## Abstract

**Background:** Chronic obstructive pulmonary disease (COPD) is a progressive lung disease characterized by significant physical and psychological burdens. However, the influence of the disease’s severity on psychological factors and functional outcomes remains unclear. This study aimed to investigate the impact of disease severity on psychological factors and functional outcomes in patients with moderate and severe COPD. **Methods:** This cross-sectional study included 98 patients with moderate (*n* = 44) or severe (*n* = 54) COPD. Anxiety and depression, guilt and shame, self-compassion, self-efficacy (PRAISE), and fear of negative evaluation were assessed. Functional capacity was evaluated with the six minute walk test (6MWT), and disease impact was assessed via the COPD assessment test (CAT). Lung function was measured through post-bronchodilator spirometry. **Results**: Compared with those with moderate COPD, those with severe COPD presented significantly greater levels of guilt (12 vs. 10; *p* < 0.01), anxiety (10 vs. 6.5; *p* < 0.01), and depression (7.5 vs. 6; *p* = 0.06). Self-compassion was significantly lower in the severe group (3.16 vs. 3.41; *p* < 0.01), whereas shame and fear of negative evaluation scores were similar between the groups. The functional capacity was significantly reduced in patients with severe COPD (217.04 ± 70.16 m vs. 286.46 ± 77.92 m; *p* < 0.01). Disease impact and dyspnea (CAT, mMRC) were worse in severe cases (*p* < 0.01). **Conclusions:** Patients with severe COPD presented significantly greater levels of guilt, anxiety, and depression, alongside lower self-compassion, worse functional outcomes, and poorer health-related quality of life, compared to those with moderate COPD.

## 1. Introduction

Chronic obstructive pulmonary disease (COPD) is the third leading cause of death worldwide, responsible for approximately 3.2 million deaths annually, and its prevalence is increasing [1]. In addition to its impact on mortality, COPD generates a significant economic burden, increasing healthcare costs and reducing work productivity [2]. Despite these serious consequences, COPD remains frequently underdiagnosed, leading to late initiation of treatment and poor disease management [2]. Dyspnea, chronic cough, and exercise intolerance are the hallmark symptoms that diminish physical function and significantly impair health-related quality of life (HRQoL) [3].

While the physical features of COPD are well described, psychological factors such as anxiety, depression [4], and self-efficacy [5] play a significant role in COPD management, influencing symptom perception, treatment adherence, and overall patient well-being [6].

Anxiety and depression are highly prevalent among patients with COPD, with reported rates ranging from 30% to 50%, particularly in those with advanced disease stages [7]. Psychological constructs play a crucial role in COPD management. Self-efficacy, defined as an individual’s belief in their ability to manage health-related challenges, is associated with greater adherence to pulmonary rehabilitation and improved symptom management [8]. Patients with higher self-efficacy are more likely to engage in physical activity and maintain disease self-management behaviors, which correlate with better functional outcomes [5]. On the other hand, self-compassion, characterized by self-kindness and emotional resilience, has been linked to reduced psychological distress and improved coping mechanisms in chronic disease management [9]. Guilt and shame are particularly relevant due to the strong link between COPD and smoking history, leading many patients to experience self-blame, stigma, and avoidance behaviors that may reduce healthcare-seeking and adherence to treatment [10]. Conversely, self-compassion, characterized by self-kindness and emotional resilience, has been associated with improved psychological well-being, greater acceptance of disease, and increased engagement in pulmonary rehabilitation [9].

Despite the emerging evidence regarding the importance of these factors, few comprehensive studies have considered how these factors interact with disease severity and functional outcomes. Addressing this gap may contribute to holistic and patient-centered interventions addressing physical and psychological well-being in COPD management.

The study aims to assess the impact of COPD severity on psychological (anxiety, depression, guilt, shame, self-compassion, self-efficacy) and functional factors, investigating the relationships between these parameters to identify how disease progression influences both the emotional burden and physical limitations of patients.

## 2. Materials and Methods

### 2.1. Study Design

We used a cross-sectional analysis to investigate the influence of disease severity on the inter-relationship between psychological factors and functional outcomes in patients with moderate and severe COPD. We followed the STROBE checklist to ensure that our reporting was complete and transparent [11].

### 2.2. Participant Recruitment

Patients with moderate and severe COPD were consecutively recruited from those seeking treatment at the Pulmonary Rehabilitation Center, Clinical Hospital of Infectious Diseases and Pulmonology, ”Victor Babes”, in Timisoara, from March 2024 to February 2025. All participants were informed about the study objective and procedures. Written informed consent for publication was obtained from each participant before enrollment. The Ethics Committee of the Clinical Hospital of Infectious Diseases and Pulmonology, ”Victor Babes”, approved the study protocol (2045/11.03.2024). We included patients with COPD if they met the following criteria: an age over 40 years; a smoking history of more than 20 pack-years; a disease severity classified as moderate or severe, as determined by spirometry (FEV_1_/FVC < 0.70 and FEV_1_ between 50 and 79% for moderate COPD, and FEV_1_ between 30 and 49% for severe COPD), a modified Medical Research Council (mMRC) dyspnea scale score ≥ 2, and a physician’s diagnosis of COPD [12]; ability to understand and complete the study questionnaires; and willingness to provide informed consent. Patients were excluded if they had other major respiratory diseases in addition to COPD, such as asthma, pulmonary fibrosis, or lung cancer; COPD exacerbation within the last 3 months, as this could impact psychological and physical assessments; a diagnosis of a severe cognitive impairment (e.g., advanced dementia or significant memory loss) or neurological disorder (e.g., Parkinson’s disease or stroke); or severe or unstable comorbidities, such as advanced heart failure (NYHA class III or IV), end-stage renal disease, or debilitating musculoskeletal disorders (e.g., severe osteoarthritis or recent fractures) that limit physical mobility.

### 2.3. Data Collection

Data were collected from participants during scheduled clinic visits between 2024 and 2025. Upon arrival, participants were screened for eligibility on the basis of the inclusion and exclusion criteria. Eligible participants were provided with a detailed explanation of the study, and then completed questionnaires assessing psychological factors. Following the psychological assessments, functional data were collected.

### 2.4. Outcomes

#### Lung Volume

We evaluated lung function using COSMED Quark PFT equipment, following the American Thoracic Society and European Respiratory Society (ATS/ERS) guidelines [13]. The forced vital capacity (FVC), forced expiratory volume in one second (FEV_1_), and modified Tiffeneau–Pinelli index (FEV_1_/FVC) were recorded post-bronchodilator.

### 2.5. Anxiety and Depression

We evaluated anxiety and depression levels with the Hospital of Anxiety and Depression scale (HADS), which is a self-report questionnaire of 14 items on a 4-point Likert scale (ranging from 0 to 3). With seven items for each subscale, it assesses anxiety and depression. The sum of all 14 items determines the overall score, whereas the sum of the 7 items for each subscale (with a range from 0 to 21) determines the score for each subscale [14]. Scores between 8 and 10 indicate moderate symptoms, whereas a score of 11 and above denotes a substantial number of symptoms that correspond with a clinical diagnosis [14].

### 2.6. Impact of the Disease

The COPD assessment test (CAT) was used to measure the impact of the disease. CAT is a self-administered questionnaire that includes eight items, each focusing on different aspects of the patient’s health related to the disease, such as cough, phlegm, chest tightness, breathlessness, activity limitation, confidence, sleep, and energy levels [15]. Each item is scored on a scale from 0 (no impact) to 5 (severe impact), resulting in a total score ranging from 0 to 40. Higher scores indicate a greater impact of COPD on patients’ health [15].

### 2.7. Shame and Guilt

Shame and guilt are crucial emotions that regulate individuals’ interactions with the surrounding environment, social relations, and self-development. The State Shame and Guilt Scale (SSGS) is a psychological tool used to measure the experience of shame and guilt [16]. We used the SSGS with ten items divided into two subscales, five for shame and five for guilt. Each item is rated on a 5-point Likert scale ranging from 1 (not feeling this way at all) to 5 (feeling this way very strongly). Subscale scores are calculated by adding together the responses for each item. Higher scores indicate stronger feelings of shame and/or guilt [16].

### 2.8. Self-Efficacy

Self-efficacy refers to an individual’s belief in his or her ability to perform specific tasks or behaviors successfully, to achieve desired outcomes [5]. Self-efficacy influences motivation, effort, persistence, and resilience in facing challenges. We used the Pulmonary Rehabilitation Adapted Index of Self-Efficacy (PRAISE) to evaluate self-efficacy [8]. It is a 15-item tool comprising ten general self-efficacy questions and five rehabilitation-specific self-efficacy questions. Each item is rated on a scale from one to five, with higher scores indicating greater self-efficacy. The PRAISE tool has demonstrated predictive validity, and can help to identify individuals with high self-efficacy, who are more likely to achieve significant health behavior changes [8].

### 2.9. Fear of Negative Evaluation

Fear of negative evaluation refers to an individual’s distress about being judged unfavorably by others [17]. It is a psychological construct associated with social anxiety, and involves concerns about incompetence, inadequacy, or unworthiness in social or evaluative situations. The Brief Fear of Negative Evaluation Scale (BFNE) is used to determine the degree of anxiety about the possibility of hostile or critical judgment by others [18]. It consists of 12 items that focus on being evaluated negatively by others. Each item is rated on a 5-point Likert scale ranging from 1 (not at all characteristic of me) to 5 (extremely characteristic of me). The scores are summed, and can vary from 12 to 60, with higher scores indicating greater fear of negative evaluation [18].

### 2.10. Self-Compassion

Self-compassion refers to the ability to treat oneself with kindness, understanding, and support during times of difficulty, failure, or personal shortcomings [19]. It is an emotionally positive self-attitude that can protect against the negative consequences of self-judgment, isolation, and rumination (such as depression) [19]. We used the Self-Compassion Scale—Short Form (SCS-SF) developed by Kristin Neff to assess individuals’ level of self-compassion [19]. It is composed of 12 items, and each item is scored on a 5-point Likert scale, from 1 (almost never) to 5 (almost always). It assesses self-compassion through six core components: self-kindness, self-judgment, common humanity, isolation, mindfulness, and overidentification. Higher scores indicate greater self-compassion.

### 2.11. Dyspnea Assessment

The Modified Medical Research Council (mMRC) Dyspnea Scale is a widely used tool for assessing the degree of breathlessness experienced by individuals with chronic respiratory diseases [20]. It consists of five grades (0–4), each describing a specific level of breathlessness related to physical activities: Grade 0—I only get breathless with strenuous exercise; Grade 1—I get short of breath when hurrying on level ground or walking up a slight hill; Grade 2—I walk slower than people of the same age on level ground because of breathlessness, or have to stop to breathe when walking at my own pace; Grade 3—I stop breathing after walking approximately 100 m or after a few minutes on level ground; Grade 4—I am too breathless to leave the house, or get breathless when dressing or undressing [20].

### 2.12. Functional Capacity

The 6 min walk test (6MWT) was performed to assess functional capacity in COPD patients, following the American Thoracic Society (ATS) guidelines [21]. The 6MWT measures the distance a person can walk comfortably on a flat, hard surface in 6 min. The test was conducted indoors, in a temperature-controlled environment, along a 30 m straight corridor marked at regular intervals, and the total distance walked was recorded in meters. For patients with COPD, a distance of <350 m often indicates severe functional limitations and a poor prognosis [22].

### 2.13. Statistical Analysis

The analysis was performed via a licensed version of MedCalc^®^ Statistical Software, version 23.0.9 64-bit (MedCalc Software Ltd., Ostend, Belgium; https://www.medcalc.org; 2024). The Shapiro-Wilk test was applied to evaluate the distribution of the data.

We conducted a power analysis using an independent samples t-test framework, assuming a moderate effect size (Cohen’s d = 0.5), a significance level of α = 0.05, and a power of 0.8 (80%). Because a stronger difference between groups was expected, the required sample sizes were 40 for moderate COPD and 50 for severe COPD.

For parametric variables, the arithmetic mean and standard deviation (SD) were used as measures of central tendency, whereas medians and interquartile ranges [IQRs] were reported for nonparametric data. Comparisons between groups were conducted via the independent samples t-test or the Mann-Whitney U test for independent data. Differences in categorical variables were assessed via the chi-square test.

The Wilcoxon signed-rank test was employed to compare two related samples or repeated measurements within the same sample, with a focus on differences in population mean ranks.

Correlation analysis was used to evaluate the strength and direction of relationships between data from COPD patients and all analyzed parameters. The Pearson correlation coefficient (r) was calculated. Statistical significance was defined as a *p*-value < 0.05.

## 3. Results

The patients in the moderate COPD group had a mean age of 65 years, with confidence intervals ranging from 62.5 to 69.5 years. In contrast, the severe COPD group had a slightly older mean age of 67 years, with a confidence interval ranging from 62 to 71 years. The p-value associated with this age difference was 0.46, indicating that there was no statistically significant difference between the two groups in terms of age. The sex distribution revealed that in the moderate COPD group, there were 28 males, constituting 63.63% of the group, whereas in the severe COPD group, there were 38 males, accounting for 70.37% of the cohort. The *p*-value for sex distribution was 0.56, suggesting that the difference in male prevalence between the two groups was not statistically significant.

The average height of the individuals in the moderate COPD group was 169.11 cm, with a standard deviation of ±6.39 cm. In contrast, the severe COPD group averaged 167.14 cm, with a standard deviation of ±7.27 cm. The p-value for height was 0.15, indicating that there was no significant difference in height between the two groups. When examining weight, the moderate COPD group had an average weight of 72.75 kg, with a standard deviation of ±10.38 kg. The severe COPD group averaged 73.29 kg, with a larger standard deviation of ±17.40 kg. The p-value for weight was 0.84, indicating that weight differences were not statistically significant. Finally, the body mass index (BMI) was reported to be 25.48 kg/m² (±3.79) for the moderate COPD group and 26.21 kg/m² (±6.03) for the severe COPD group, with a p-value of 0.46, which again suggests that there was no significant difference in BMI between these groups. No significant difference was observed in the group distribution. A comparison of the analyzed parameters between the two groups is presented in Table 1.

The data presented for the COPD groups are expressed as values (percentages), arithmetic means ± SDs, or medians and [IQRs]. BMI—body mass index; PRAISE—Pulmonary Rehabilitation Adapted Index of Self-Efficacy; BFNE—Brief Fear of Negative Evaluation; SCS-SF—Self-Compassion Scale—Short Form; SSGS—State Shame and Guilt Scale; HADS—Hospital of Anxiety and Depression Scale; CAT—COPD Assessment Test; mMRC—Modified Medical Research Council; 6MWT—six minute walk test; FVC—forced vital capacity; FEV_1_—forced expiratory volume in one second.

The analysis revealed significant psychological, emotional, and physical differences between the two groups. The moderate COPD group presented significantly higher PRAISE scores (52 vs. 46.5; *p* < 0.01). However, BFNE scores, which assess fear of negative evaluation, were not significantly different between the groups (27.5 ± 6.25 vs. 27.9 ± 6.55; *p* = 0.75).

With respect to self-compassion, several subscales showed notable differences. The moderate COPD group scored higher for self-judgment (3.5 vs. 3; *p* = 0.04) and common humanity (3 vs. 3; *p* = 0.02). Moreover, the moderate COPD group exhibited higher levels of isolation (3.5 vs. 3; *p* < 0.01) and mindfulness (4 vs. 3; *p* < 0.01). The total SCS-SF score was also significantly greater in the moderate COPD group (3.41 vs. 3.16; *p* < 0.01), reflecting greater overall self-compassion in this cohort.

Emotional measures, such as guilt and shame, also differed. The severe COPD group displayed higher levels of guilt (12 vs. 10; *p* < 0.01), whereas the shame scores did not significantly differ between the groups (10 vs. 9; *p* = 0.46). Overall emotional distress, measured by the SSGS, was greater in the severe COPD group (22.5 vs. 20; *p* = 0.01). Anxiety and depression scores were also significantly elevated in patients with severe COPD (10 vs. 6.5; *p* < 0.01 and 7.5 vs. 6; *p* = 0.06, respectively), as was the total HADS score (16 vs. 13; *p* < 0.01).

Severe COPD patients had worse scores on the CAT (24.42 ± 5.18 vs. 20.77 ± 5.31; *p* < 0.01) and mMRC dyspnea scales (3 vs. 2; *p* < 0.01). The functional capacity, measured by the 6MWT, was significantly lower in the severe COPD group, in terms of both distance (217.04 ± 70.16 m vs. 286.46 ± 77.92 m; *p* < 0.01) and percentage (39.74% vs. 65.5%; *p* < 0.01).

Lung function tests also revealed significant impairments in patients with severe COPD. FVC and FEV_1_ values, both in liters and as percentages, were markedly lower in this group (*p* < 0.01 for all). Additionally, the FEV_1_/FVC ratio was significantly lower in this group (0.49 ± 0.9 vs. 0.66 ± 0.3; *p* < 0.01), suggesting more significant respiratory dysfunction.

In patients with moderate COPD, significant correlations were observed between anxiety and the 6MWT distance (r = −0.32, *p* = 0.03), depression and FVC (r = −0.35, *p* = 0.01), the CAT score and the 6MWT distance (r = −0.54, *p* < 0.01), the mMRC score and the 6MWT percentage (r = −0.46, *p* < 0.01), and the PRAISE score and the 6MWT distance (r = 0.34, *p* = 0.02). Depression and CAT scores showed a weaker, nonsignificant correlation (r = 0.21, *p* = 0.08).

**Figure 1:**
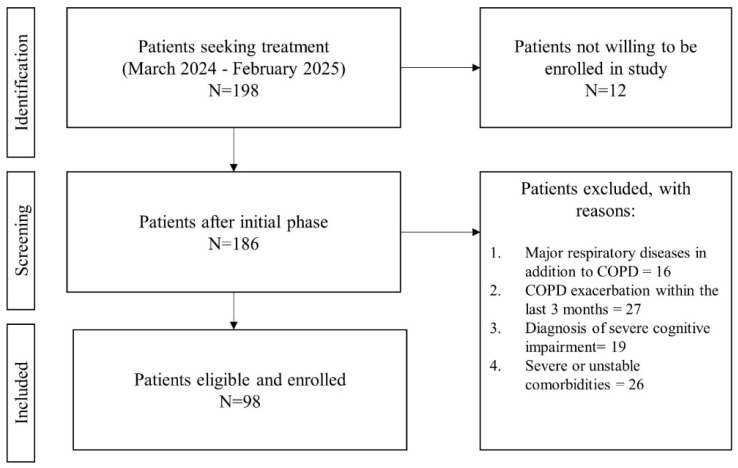
Flowchart of patient selection process.

**Figure 2:**
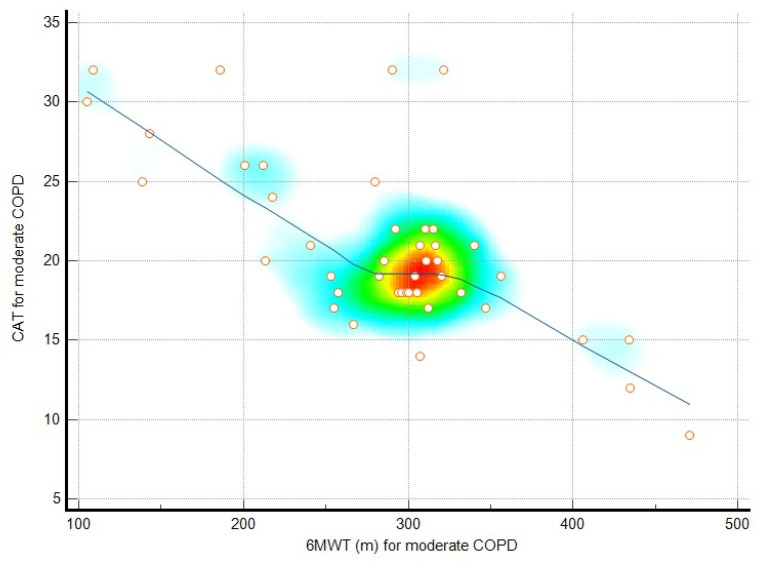
A scatter plot with density shading, illustrating a negative correlation between CAT and 6MWT, as shown by the downward-sloping blue regression line. Individual data points, represented as orange circles, are distributed across the plot, with a density gradient (from blue to red) highlighting regions with higher concentrations of points. The densest area, marked in red, aligns closely with the trend line, indicating a significant relationship between the variables, where one decreases as the other increases.

**Figure 3:**
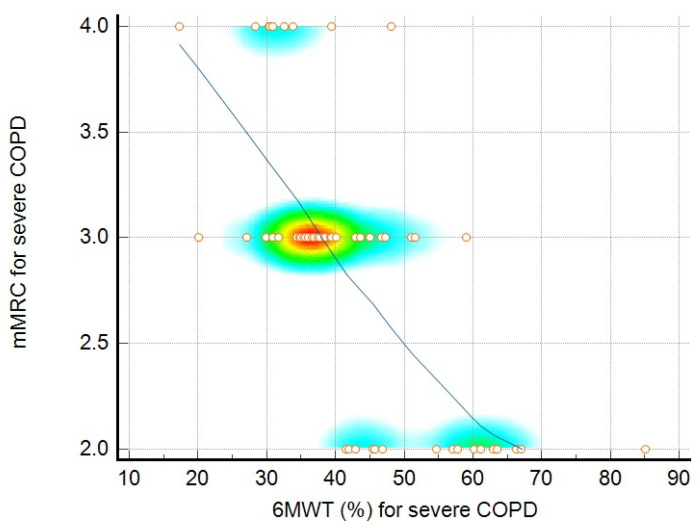
A scatter plot with density shading, illustrating a negative correlation between mMRC and 6MWT (%), as shown by the downward-sloping blue regression line. Individual data points, represented as orange circles, are distributed across the plot, with a density gradient (from blue to red) highlighting regions with higher concentrations of points. The densest area, marked in red, aligns closely with the trend line, indicating a significant relationship between the variables, where one decreases as the other increases.

In patients with severe COPD, significant correlations were found between depression and FVC (r = −0.39, *p* = 0.01), the CAT score and the 6MWT distance (r = −0.45, *p* < 0.01), the mMRC score and the 6MWT percentage (r = −0.70, *p* < 0.01), and the PRAISE score and the 6MWT distance (r = 0.26, *p* = 0.04). Anxiety and the 6MWT distance were not significantly correlated (r = −0.23, *p* = 0.07). Depression and CAT scores were significantly correlated (r = 0.34, *p* = 0.01). The relationships among the analyzed parameters are presented in Table 2. Figure 1 presents the relationship between the CAT score and the 6MWT distance (m) for moderate COPD patients. Figure 2 presents the relationship between the mMRC score and the 6MWT distance (%) for patients with severe COPD. Figure 3 represents a scatter plot with density shading, illustrating a negative correlation between mMRC and 6MWT (%).

## 4. Discussion

The relationships between psychological factors and functional outcomes in COPD patients are both complex and multifaceted. Our results emphasize the strong association between psychological distress and diminished functional performance. Anxiety and depression, as measured by the HADS, were significantly correlated with a reduced 6MWT distance and worse scores on the CAT, particularly in patients with severe COPD. Yohannes et al. identified a bidirectional relationship between psychological distress and physical symptoms, with distress exacerbating the perception of breathlessness and fatigue, leading to further inactivity and deterioration [4]. Disease-specific anxiety plays a prominent role in shaping functional limitations. Von Leopoldt and Janssens highlighted that fear of breathlessness often leads to activity avoidance, exacerbating physical deconditioning and reinforcing a cycle of inactivity, thereby worsening symptoms [23].

Depression, as assessed by the depression subscale of the HADS, was another significant predictor of poorer functional outcomes, with its impact being more pronounced in the severe COPD group. The association between depressive symptoms and reduced 6MWT performance aligns with the findings of Volpato et al., who emphasized that depression is a significant barrier to engagement in pulmonary rehabilitation [24]. Higher depression scores in our severe COPD group were also correlated with higher CAT scores, highlighting the accumulated burden of psychological and physical challenges in the advanced stages of the disease. The stronger negative correlations between anxiety, depression, and functional outcomes in severe COPD suggest that a greater disease burden, increased dyspnea perception, and further physical limitations contribute to heightened psychological distress. In moderate COPD, patients may still retain more functional reserve and adaptive coping mechanisms, potentially buffering these effects.

As measured by the PRAISE questionnaire, self-efficacy was positively correlated with functional outcomes across the moderate and severe COPD groups. This could highlight the role of self-efficacy in enabling patients to maintain physical performance, even in the face of disease-related limitations. This aligns with the findings of Liacos et al., who demonstrated that higher baseline PRAISE scores were associated with reduced sedentary time following pulmonary rehabilitation [8]. Similarly, a review by Yi et al. highlighted self-efficacy as a critical component of chronic disease management. Self-efficacy was associated with better adherence to pulmonary rehabilitation programs, improved treatment compliance, and enhanced quality of life [5]. In our study, the relationship between self-efficacy and functional outcomes was particularly evident in patients with moderate COPD, with higher PRAISE scores strongly correlated with better performance on the 6MWT.

Shame and guilt are self-conscious emotions that are frequently experienced by patients with COPD, especially those who attribute their condition to perceived personal failure, and they are linked to poorer psychological well-being and quality of life [9,10]. While guilt levels were significantly higher in the severe COPD group, shame scores did not differ between groups. This distinction may arise from the different psychological mechanisms underlying these emotions. Guilt is often linked to behavioral self-attribution, such as smoking history and perceived responsibility for disease progression, which may intensify as COPD worsens [25]. In contrast, shame is more closely tied to social identity and stigma, which may remain relatively stable regardless of disease severity [25]. Compared with Harrison et al., we observed that shame and guilt scores were significantly lower in the studied population. This discrepancy could be attributed to how self-conscious emotions are perceived across populations. A more recent study revealed that patients with severe COPD experience more guilt and shame than mild and moderate disease patients do, with no differences in the time since diagnosis or number of comorbidities [25].

Self-compassion has emerged as a novel factor influencing resilience in COPD patients. In our study, the self-compassion score was significantly lower in patients with severe COPD than in moderate COPD patients. These findings could indicate a decline in psychological resilience with disease progression. The results of the SCS-SF score were higher than those reported by Harrison et al., potentially reflecting cultural and demographic differences and the fact that the authors did not stratify self-compassion by severity, making it difficult to compare trends across severity levels directly. Benzo et al. investigated the associations between self-compassion, quality of life, and self-management ability in 310 individuals with moderate-to-severe COPD [26]. The authors reported that self-compassion is significantly associated with self-management skills and quality of life, regardless of disease severity [26]. Our study revealed a decline in self-compassion with increasing disease severity, highlighting the need for stage-specific interventions to improve psychological resilience in COPD patients.

Fear of negative evaluation, in the context of COPD, can arise from visible symptoms such as coughing, shortness of breath, or the use of medical devices such as oxygen tanks, leading to feelings of shame and stigma [10]. Our study highlights the role of social anxiety, as measured by BFNE, in the management of COPD. While other authors have reported higher BFNE scores [9], our results indicate a consistent fear of negative evaluation across disease severities. Our findings suggest that social anxiety is a common issue in COPD patients, independent of disease progression. Fear of negative evaluation is a psychological construct that is not well studied in this population, and should be addressed through interventions to increase social confidence. The lack of differences in BFNE scores and shame could indicate that social anxiety and self-conscious emotions may not be directly influenced by COPD severity, but rather by other psychological or social factors. Future studies should explore these aspects further.

When interpreting our data, readers should consider that the study’s cross-sectional design limits its ability to establish causal relationships between psychological factors, disease severity, and functional outcomes. The exclusion of other COPD groups and recent exacerbations limits the applicability of the findings to other broader COPD populations. The reliance on self-report questionnaires may have introduced bias, and cultural and socioeconomic differences should be considered when interpreting emotions such as guilt or shame.

## 5. Conclusions

Patients with severe COPD presented significantly greater levels of guilt, anxiety, and depression, alongside lower self-compassion, worse functional outcomes, and poorer health-related quality of life, than did those with moderate COPD. In contrast, shame and fear of negative evaluation were similar between the groups, indicating consistent self-conscious emotions, regardless of disease severity.

## Figures and Tables

**Table 1:** A comparison of the baseline characteristics and analyzed parameters between the two groups.

Parameters	Moderate COPD (*n* = 44)	Severe COPD (*n* = 54)	*p*-Value
Age (years)	65; (62.5, 69.5)	67; (62, 71)	0.46
Male gender	28 (63.63%)	38 (70.37)	0.56
Height (cm)	169.11 ± 6.39	167.14 ± 7.27	0.15
Weight (kg)	72.75 ± 10.38	73.29 ± 17.40	0.84
BMI (kg/m^2^)	25.48 ± 3.79	26.21 ± 6.03	0.46
Comorbidities	1.8 ± 0.9	3.4 ± 1.2	<0.01
PRAISE	52; (47, 55)	46.5; (39, 54)	<0.01
BFNE	27.5 ± 6.25	27.9 ± 6.55	0.75
Self-kindness	3.5; (3, 4)	4; (3, 4.5)	0.56
Self-judgment	3.5; (3, 4)	3; (2, 3.5)	0.04
Common humanity	3; (3, 4)	3; (2.5, 3.5)	0.02
Isolation	3.5; (3, 4)	3; (2.5, 3)	<0.01
Mindfulness	4; (3, 4)	3; (2.5, 3.5)	<0.01
Overidentification	3; (3, 3.5)	3.5; (2.5, 4)	0.84
Total SCS-SF	3.41; (3.33, 3.58)	3.16; (2.91, 3.41)	<0.01
Shame	9; (6.5, 11)	10; (6, 12)	0.46
Guilt	10; (8, 12)	12; (10, 16)	< 0.01
Total SSGS	20; (16, 22)	22.5; (17, 26)	0.01
Anxiety	6.5; (5.5, 7.5)	10; (7, 12)	<0.01
Depression	6; (5, 7)	7.5; (4, 9)	0.06
Total HADS	13; (10.5, 15)	16; (13, 20)	<0.01
CAT	20.77 ± 5.31	24.42 ± 5.18	<0.01
mMRC	2; (2, 2)	3; (2, 3)	<0.01
6MWT (m)	286.46 ± 77.92	217.04 ± 70.16	<0.01
6MWT (%)	65.5; (56.5, 70)	39.74; (34.74, 51.02)	<0.01
FVC (L)	2.72 ± 0.31	2.29 ± 0.43	<0.01
FVC (%)	73.31 ± 3.97	66.41 ± 8.69	<0.01
FEV_1_ (L)	1.77; (1.7, 1.91)	1.09; (0.99, 1.15)	<0.01
FEV_1_ (%)	55; (54, 58)	34; (31, 38)	<0.01
FEV_1_/FVC	0.66 ± 0.3	0.49 ± 0.9	<0.01

**Table 2:** Relationships between the analyzed parameters of the two groups.

Parameters	r/rho for Moderate COPD (*n* = 44)	*p*-Value	r/rho for Severe COPD (*n* = 54)	*p*-Value
Anxiety–6MWT (m)	−0.32	0.03	−0.23	0.07
Depression–FVC (L)	−0.35	0.01	−0.39	0.01
CAT–6MWT (m)	−0.71	<0.01	−0.45	<0.01
mMRC–6MWT (%)	−0.46	<0.01	−0.70	<0.01
PRAISE–6MWT	0.34	0.02	0.26	0.04
Depression–CAT	0.21	0.08	0.34	0.01

6MWT—Six minute walk test; FVC—forced vital capacity; mMRC–Modified Medical Research Council; PRAISE—Pulmonary Rehabilitation Adapted Index of Self-Efficacy; CAT—COPD Assessment Test.

## Data Availability

The supporting data for the findings of this study can be obtained by contacting the corresponding author upon request. However, the data cannot be publicly accessed, due to privacy and ethical considerations.

## Abbreviations

The following abbreviations are used in this manuscript:

COPD	Chronic obstructive pulmonary disease
HRQoL	Health-related quality of life
FEV1	Forced expiratory volume in 1 s
FVC	Forced vital capacity
mMRC	Modified Medical Research Council scale
ATS	American Thoracic Society
ERS	European Respiratory Society
HADS	Hospital Anxiety and Depression Scale
CAT	COPD assessment test
SSGS	State Shame and Guilt Scale
PRAISE	Pulmonary Rehabilitation Adapted Index of Self-Efficacy
BFNE	Brief Fear of Negative Evaluation
SCS-SF	Self-Compassion Scale—Short Form
6MWT	Six Minute Walk Test
SD	Standard deviation
IQRs	Interquartile ranges
BMI	Body mass index
